# Data on airborne bacteria and fungi emission from a conventional hospital wastewater treatment plant

**DOI:** 10.1016/j.dib.2019.105019

**Published:** 2019-12-18

**Authors:** Ahmadreza Yazdanbakhsh, Mona Ghazi, Fatemeh Sahlabadi, Fahimeh Teimouri

**Affiliations:** a Environmental and Occupational Hazards Control Research Center, Shahid Beheshti University of Medical Sciences, Tehran, Iran; b Department of Microbiology, School of Medicine, Shahid Beheshti University of Medical Sciences, Tehran, Iran; c Students’ Research Committee, Shahid Beheshti University of Medical Sciences, Tehran, Iran; d Environmental Science and Technology Research Center, Department of Environmental Health Engineering, Shahid Sadoughi University of Medical Sciences, Yazd, Iran

**Keywords:** Hospital, Wastewater treatment plant, Bioaerosol, Microbial diversity, Particle concentration

## Abstract

The lack of necessary air pollution control measures in the construction of hospital wastewater treatment plants results in the release of harmful bioaerosols in and around the hospital. A sampling of airborne bacteria and fungi was performed using the gravitational method in 9 sites including an upwind site, intra-plant and outside a hospital wastewater treatment plants with activated sludge technology in Tehran (1, 5 and 3 points, respectively) from March to June. Bioaerosol on nutrient agar media were identified quantitatively and qualitatively. Intra-plant airborne particulate matter concentrations were measured by an optical particle sizer in intervals of 6 s for 60 min. The environmental parameters were also recorded in the sampling period. Experimental data was collected and analyzed by Excel software and SPSS statistical software version 23, respectively. This work is useful to help manage bioaerosols exposure risk such as WWTP.

**Table:** Specifications Table

Subject	Environmental Sciences
Specific subject area	Air pollution and bioaerosol
Type of data	Figures
How data were acquired	Bacterial colonies were initially characterized according to their staining characteristics, morphology, and microscopic examination. Then, all positive cultures on the media were plated on selective media and identified further by biochemical tests. The fungal isolates were identified on the basis of microscopic (using Lactophenol cotton blue staining) and macroscopic characteristics (with the aid of an Atlas of Mycology). Airborne particulate matter concentrations were analyzed with an optical particle sizer (Grimm; Grimm Aerosoltechnik, Model 1.108, Germany) in 15 channels between <0.3 and 20 μm and intervals of 6 s. Experimental data was collected and analyzed by Excel software and SPSS statistical software version 23 (SPSS Institute, Cary, USA), respectively.
Data format	Raw and analysed
Parameters for data collection	Air sampling was carried out according to the EPA sampling guideline. The gravitational method was applied to collect bioaerosol samples in Petri dishes containing different cultures.
Description of data collection	9 sites were selected for sampling according to the type of process, wind direction and patient traffic, including an upwind site outside the plant (one point), intra-plant (five points: aeration tank, settling tank, sewage inlet to aeration tank, sewage pumping station) and outside plant (three points). Air sampling was carried out according to the EPA sampling guideline, once every 6 days for 4 months from March to June. The gravitational method was applied to collect bioaerosol samples in three open 9-cm diameter Petri dishes containing different cultures. This was performed by exposing the open lids prepared in the Petri dishes containing Sabouraud dextrose agar (SDA) for fungi with chloramphenicol added to inhibit the proliferation of bacteria, Nutrient Agar (NA) for bacteria and Agar MacConkey (Merck Germany) for Gram-negative bacteria with cycloheximide added to suppress fungal growth in a period of 60 minutes.
Data source location	Airborne bioaerosol were collected from a conventional hospital wastewater treatment plant in Tehran, Iran (Latitude: 35° 41′ 39.80″ N and Longitude: 51° 25′ 17.44″ E).
Data accessibility	Data represented with the article

**Table:** 

**Value of the Data** • The data are not only of great importance in relation to public safety of this particular WWTP, but also future WWTPs of its kind. • This data can be useful for managers and all related stakeholders working in the fields of manage microbial exposure risk involving HWWTPs. • The data can be taken into consideration in the design and maintenance of the hospital wastewater treatment plants with respect to occupational safety and health. • This data can be useful to set standards for levels of acceptable microbial population and can also be used to suggest suitable guidelines that will help to decrease microbial density in the air of around WWTPs.

## 1 Data description

**Fig. 1:**
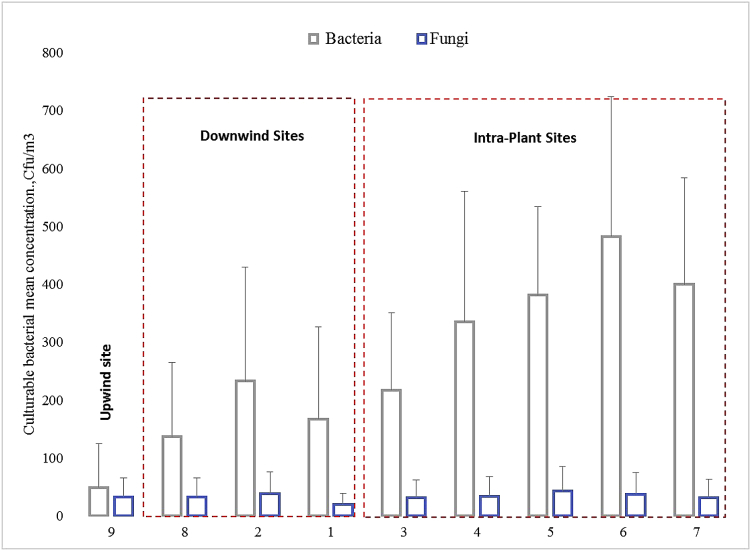
The airborne bacterial and fungal concentrations of the air samples collected from the sampling sites.

**Fig. 2:**
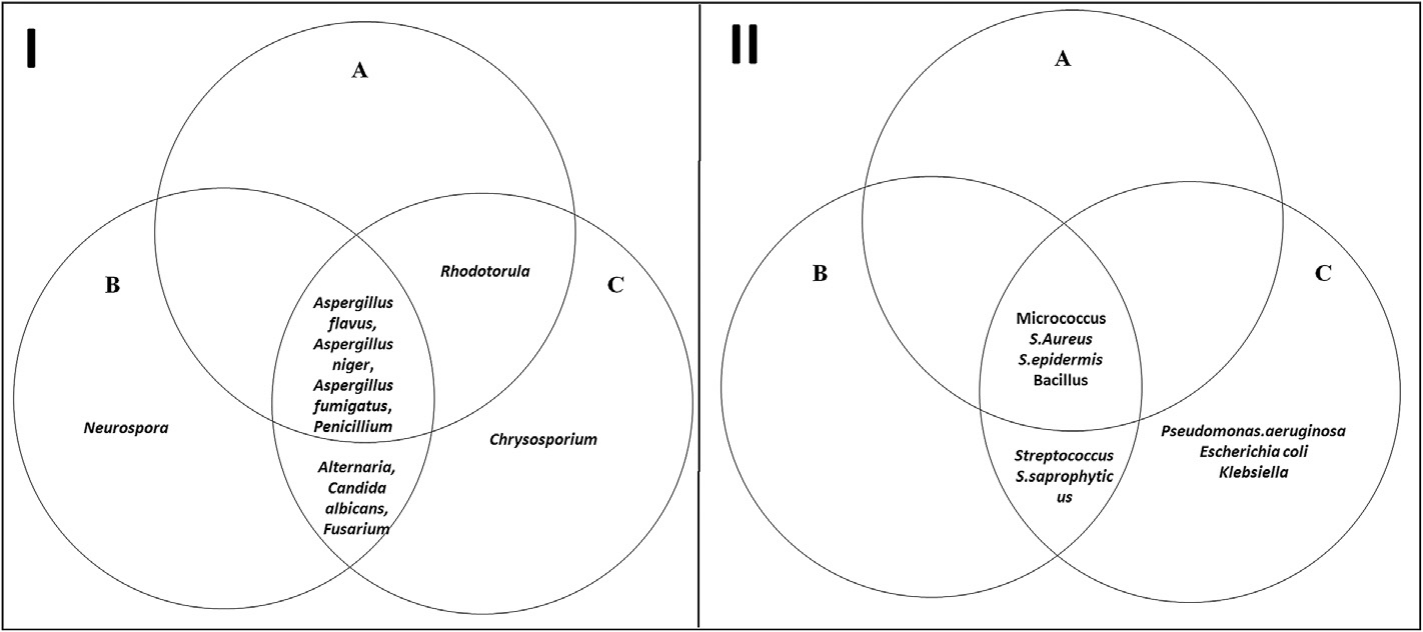
The Venn diagram showing airborne bacterial (I) and fungal (II) genera identified from the sampling sites: a) upwind the WWTP, b) downwind sites, c) intra-plant sites. Overlap presents genera detected in multiple sites.

**Fig. 3:**
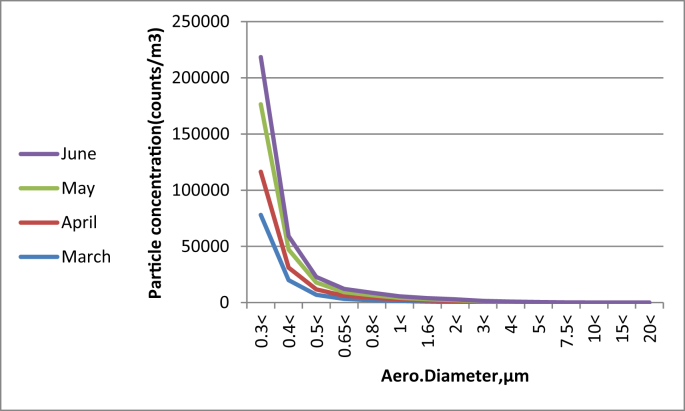
Particle size distributions identified using an optical particle sizer at different months.

**Fig. 4:**
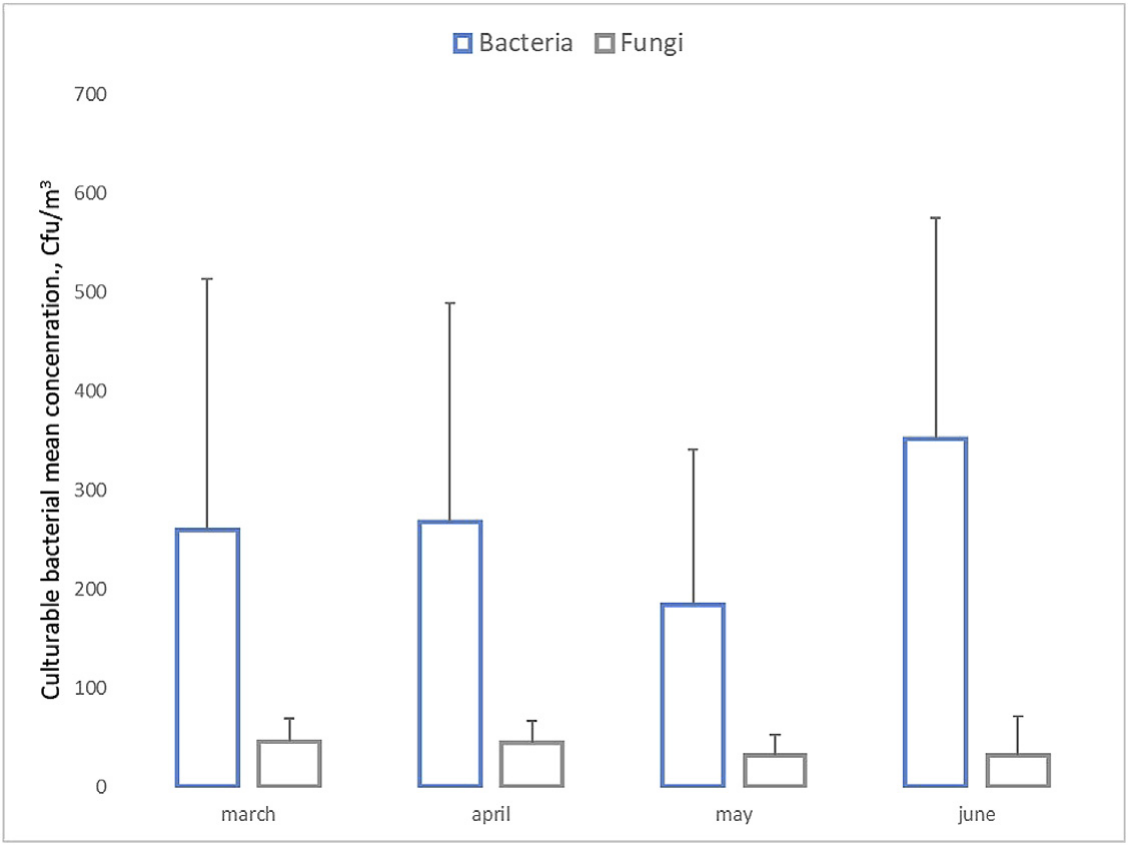
The airborne bacterial and fungal concentrations of the air samples collected during the four months of sampling.

The current dataset contains 4 figures. The concentration of airborne bacteria and fungi in sampling sites is shown in Fig. 1. Bioaerosols genera and some of their species detected in the collected air samples is presented in Fig. 2. Fig. 3 shows the distribution of the particle concentration and size in five locations intra-plant. The airborne bacterial and fungal concentrations during the four months of sampling is shown in Fig. 4.

## 2 Experimental design, materials, and methods

### 2.1 Plant description and sampling locations

**Fig. 5:**
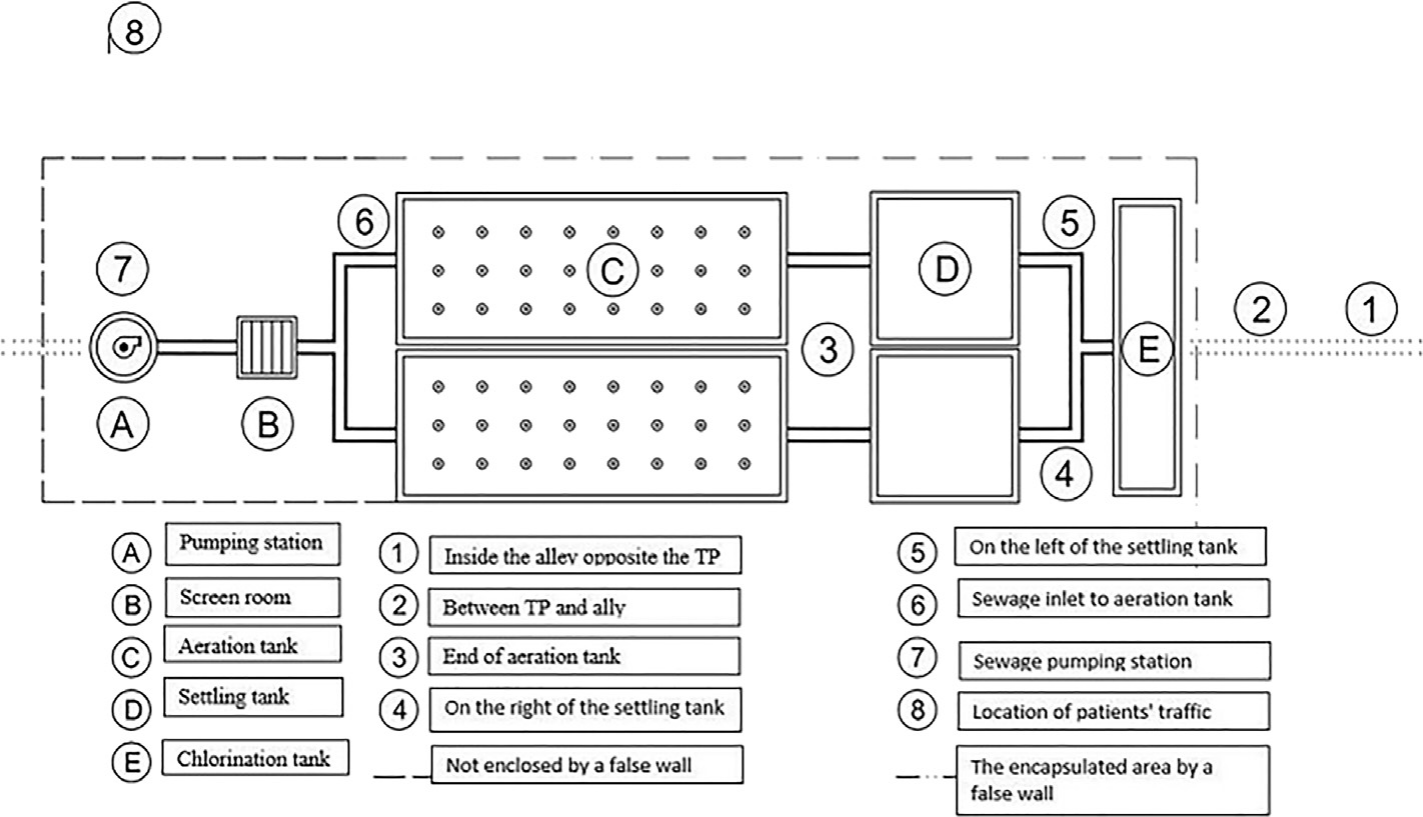
Sampling locations of Hospital wastewater treatment plant.

The work was performed at the WWTP of a hospital (design flow: 800 m^3^/day). The treatment plant covers an area of 465 m^2^. The main note is the location of treatment that close to residential area (50 m) of the east part of Tehran, the capital of Iran country (Fig. 5).

Influent sewage from all parts of the hospital is pumped to the screen room prior to the biological treatment. The secondary treatment is based on activated sludge and diffuser aeration system. In this work, 9 sites were selected for sampling according to the type of process, wind direction and patient traffic, including an upwind site outside the plant (one point), intra-plant (five points: aeration tank, settling tank, sewage inlet to aeration tank, sewage pumping station) and outside plant (three points) as shown in Fig. 5.

### 2.2 The sampling method

Sampling was carried out according to the EPA sampling guideline, once every 6 days for 4 months from March to June. The gravitational method was applied to collect bioaerosol samples in three open 9-cm diameter Petri dishes containing different cultures. This was performed by exposing the open lids prepared in the Petri dishes containing Sabouraud dextrose agar (SDA) for fungi with chloramphenicol added to inhibit the proliferation of bacteria, Nutrient Agar (NA) for bacteria and Agar MacConkey (Merck Germany) for Gram-negative bacteria with cycloheximide added to suppress fungal growth in a period of 60 minutes [1,2]. Twenty-four samples (36 plates) were collected from each location, and a total of 216 samples (324 plates) were obtained for bacteria and fungi. The location for plate contact was in a respiratory height of approximately 1.5 m above floor level and a distance of 1 m from the walls and obstacles (due to EPA guideline). During sampling, air temperature, relative humidity (RH), UV index, and wind speed all corresponded to the average recorded throughout the monitoring time, according to the UK meteorology report. Intra-plant airborne particulate matter concentrations were analyzed with an optical particle sizer (Grimm; Grimm Aerosoltechnik, Model 1.108, Germany) in 15 channels between <0.3 and 20 μm and intervals of 6 s. After every collection cycle, the plates were covered, kept in a tight sealed case, and immediately transferred in a cool box to the microbiology laboratory unit at the Department of Medical Sciences, Shahid Beheshti University. The plates were then incubated for 48 h at 37° C for bacteria and for 3–5 days at 25 °C for fungi. After incubation, the concentration of the growing colonies was measured as colony forming units (CFUs), and the result was recalculated per cubic meter of air (CFU/m^3^). The sedimentation method was based on Omelianski's formula [3].

$$N=5a*10^{4}{\left(b.t\right)}^{-1}$$

where N is the microbial CFU/m^3^, “a” is the number of colonies per Petri dish, “b” is the dish surface (cm^2^), and “t” is the exposure time (in minutes).

### 2.3 Detection and quantification of bacterial and fungal bioaerosols

Total bacteria and fungi were enumerated as the number of grown colonies and expressed as colony-forming units per plate unit. The identical colonies were sub-cultured into NA or SDA plates, incubated appropriately, and stored for further identification and characterization. Bacterial colonies were initially characterized according to their staining characteristics, morphology, and microscopic examination. Then, all positive cultures on the media were plated on selective media and identified further by biochemical tests [4]. Moreover, the fungal isolates were identified on the basis of microscopic (using Lactophenol cotton blue staining) and macroscopic characteristics (with the aid of an Atlas of Mycology) [5,6].

### 2.4 Data analysis

Experimental data was collected and analyzed by Excel software and SPSS statistical software version 23 (SPSS Institute, Cary, USA), respectively. One sample Kolmogorov-Smirnov test was conducted to determine the normality of the concentration of particles, bacteria, and fungi. The effect of sampling date and location was studied on the concentration using the analysis of variance (ANOVA) method. T-test analysis was performed to examine the different intra-plant and outside concentrations of airborne bacteria and fungi. The statistical significance of the correlation between environmental factors and airborne microorganisms was verified by the Pearson correlation analysis test. A p-value of 0.05 indicated a statistically significant difference.
